# Infliximab eye drops treatment in corneal neovascularization

**Published:** 2015

**Authors:** OB Voiculescu, LM Voinea

**Affiliations:** * Department of Cellular and Molecular Medicine, “Carol Davila” University of Medicine and Pharmacy, Bucharest, Romania; ** Department of Ophthalmology, University Emergency Hospital Bucharest, Romania

**Keywords:** neovascularization, antiVEGF agents

## Abstract

Corneal neovascularization is a serious condition that may arise secondary to chemical burns, ischemia, infection, trauma, and inflammation and represents a major cause of blindness. This study investigated the efficacy of topical application of infliximab [tumor necrosis factor-a (TNF-a) monoclonal antibody] in the treatment of corneal neovascularization in the rabbit model.

## Introduction

Angiogenesis is the process of new blood vessel growth from the pre-existing vascular structures. Corneal angiogenesis occurs in several pathological conditions and brings about a variety of unwanted consequences. Corneal NV can be asymptomatic, but more often it results in severe visual disorders, and in some cases, in practical blindness, because of unfavorable corneal opacification. Vascular endothelial growth factor (VEGF), one of the most important mediators of angiogenesis, is upregulated during neovascularization. TNFα tumor necrosis factor is known to be involved in neovascularization by inducing VEGF [**1**-**5**].

## Materials and method

16 rabbits weighing between 2.0 and 2.5 kg were used in this study. Before the experiment, all the rabbits were assessed for corneal abnormalities and were confirmed to have normal corneas.

Neovascularization was induced in 32 eyes of 16 rabbits by pressing a 2-mm diameter alkaline-coated applicator (NaOH 1mol/ L) after induced general anesthesia with ketamine hydrochloride 35mg/ kg and xylazine hydrochloride 2% 5mg/ kg. 

**Fig. 1:**
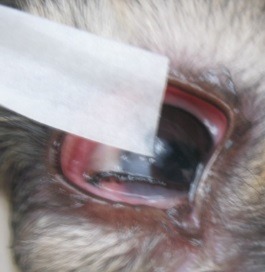
Alkaline-coated applicator (NaOH 1mol/ L)

Fourteen days after the chemical burn, corneal neovascularization was confirmed and the subjects were divided into 4 groups of 8 rabbits: no treatment control (group I), group II, III and IV were treated with Infliximab eye drops of varying concentrations (1, 2, and 4 mg/ mL) three times a day for two weeks. 100 mg of Infliximab powder (Remicade) were dissolved in 1mL of sterilized distilled water, 24 mL of BSS were added and 4 mg/ mL of eye drops were produced. Then, the 4 mg/mL of eye drops were diluted and 2 and 1 mg/ mL of eye drops were produced. The experiment was executed for 7 days after the induction of corneal neovascularization.

Digital photographs of the cornea were taken with Olympus camera E410 before initiating the treatment and after 2 weeks. The pictures were analyzed to determine the percentage area of the cornea covered by neovascularization. 

**Fig. 2:**
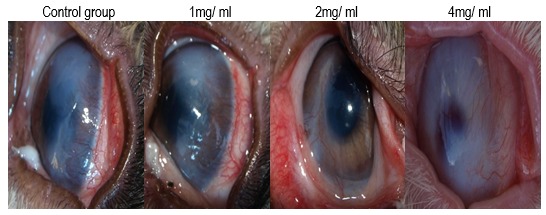
Before treatment

**Fig. 3:**
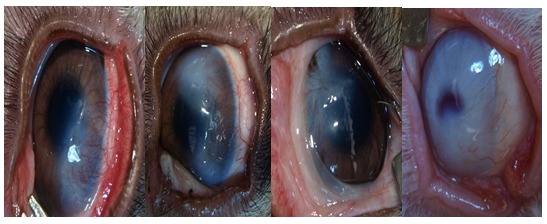
After treatment

Two weeks after the treatment, the rabbits were euthanasiated and all eyes were enucleated. Corneal sections were analyzed histopathologically. 

## Results

In digital photographs, the neovascularized area was decreased in all 3 experimental groups (1, 2, and 4 mg/ mL) compared with the control group (balanced salt solution). The median percentages of corneal neovascularization in groups 2, 3 and 4 (the study groups) were 63, 55 and 43% respectively and was significantly lower than in group 1 (72%). Histological examination showed markedly regressed new vessels and inflammatory infiltrate in treatment groups, but corneal stromal thickness and cellularity persisted. 

**Fig. 4:**
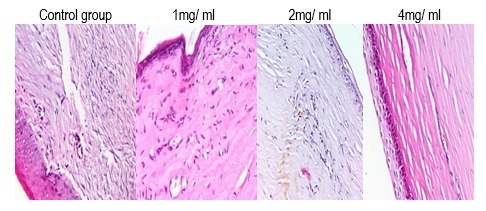
Histological examination

## Conclusions

The inflammation caused by a chemical burn can be minimized with Infliximab, which penetrates the cornea and is safe to the ocular surface. We suggested that the topical application of infliximab might be a useful treatment in ocular caustications. This study demonstrated that the topical administration of infliximab inhibits corneal neovascularization and decreases inflammation and fibroblast activity in a rabbit model of corneal neovascularization induced by alkali burn. It may provide a promising alternative for the ocular topical antiangiogenic therapy.

**Acknowledgement**

This paper was supported by the Sectoral Operational Programme Human Resources Development financed from the European Social Fund under the contract number POSDRU/159/1.5/S/137390.
